# Inline NMR Detection of Li^+^ in Aqueous Solutions Using a Cryogen-Free Magnet at 4.7 T

**DOI:** 10.3390/molecules31020267

**Published:** 2026-01-13

**Authors:** Eric Schmid, Jens Hänisch, Frank Hornung, Hermann Nirschl, Gisela Guthausen

**Affiliations:** 1 Institute of Mechanical Process Engineering and Mechanics, Karlsruhe Institute of Technology, 76131 Karlsruhe, Germany; 2 Institute for Technical Physics, Karlsruhe Institute of Technology, 76344 Eggenstein-Leopoldshafen, Germany; 3 Chair of Water Chemistry and Water Technology, Engler-Bunte-Institut, Karlsruhe Institute of Technology, 76131 Karlsruhe, Germany

**Keywords:** NMR, inline process monitoring, lithium extraction, relaxation, lithium NMR, cryogen-free magnet, mobile NMR

## Abstract

Lithium is of major importance for many areas of technology, especially batteries, and is therefore relevant to both the industrial and private sectors. High-performance, ideally inline-compatible analytics are important for economical and environmentally friendly lithium extraction. Nuclear Magnetic Resonance is an established analytical method that has already been used in numerous inline applications. For this study on ^7^Li NMR in flow, a cryogen-free magnet with a variable magnetic field was used, whereby a field strength of 4.7 T was set for the measurements for compatibility reasons. The influences of flow velocity, repetition time, and lithium concentration were investigated in spin echo measurements. This allows for defining limitations and potential fields of application for the measurement setup. In addition, the possibilities of internal pre-polarization were investigated. The results show that the method and setup are well suited for inline flow measurements on ^7^Li and have great potential for expanding the range of applications.

## 1. Introduction

Lithium has become an incredibly important raw material for numerous technologies, particularly for the production of high-performance batteries for electric mobility and mobile electronic devices [1]. The limited resources that can currently be economically exploited via mining make the development of new technologies and the exploitation of further mining areas attractive from both economic and ecological perspectives. Suitable process-compatible analysis methods are essential for powerful, efficient, and sustainable technical processes for lithium extraction. Inline methods offer advantages over offline methods in that chemical or physical measurements are available almost instantly while being non-destructive and contactless, thereby providing the basis for an efficient and agile technical process [2]. This increases lithium output and reduces energy consumption on the one hand. On the other hand, since the salt solutions are corrosive, this approach of minimum sample preparation and treatment in a closed loop leads to minimum destruction of the measurement equipment.

Nuclear Magnetic Resonance (NMR) is a widely used and well-established analytical tool with applications both in research, such as chemical structure elucidation, and in the industrial sector, for example, in quality control [3,4,5,6,7,8]. Industrial NMR applications require monitoring tools that are robust against external influences in a process plant, generate a small magnetic stray field, and are easy to operate with low maintenance needs. Classic high-field NMR instruments with their superconducting magnets cooled with liquid helium and nitrogen are often unsuitable for this purpose. Furthermore, mobile applications are hardly feasible. Instead, low-field NMR with permanent magnets is well suited for this purpose and has already been used extensively for inline applications [9,10,11,12,13,14,15]. A low-field NMR sensor for ^7^Li inline measurements on lithium-containing brines has also been developed and applied [16]. However, when measuring nuclei other than ^1^H and samples with small concentrations of the target product, a stronger magnetic field *B*_0_ should be used to increase the signal-to-noise ratio. The gyromagnetic ratio γ of ^7^Li is a factor of 2.573 smaller than that of ^1^H, the spin quantum number is 3/2, and the natural abundance is with 92.4% slightly lower than of ^1^H so that a stronger *B*_0_ with inherently larger sensitivity is desirable for the development of process-compatible applications of ^7^Li NMR [17,18,19].

In this study, ^7^Li NMR on flowing aqueous lithium chloride (LiCl) solutions was performed with a superconducting, cryogen-free magnet at a *B*_0_ of 4.7 T as a basis for the development of an inline-capable measurement method for monitoring the lithium concentration in process streams during lithium extraction. In addition to the influence of lithium concentration, the influences of flow velocity, repetition time, and polarization length were also investigated. A compact, industrial-grade Bruker minispec NF instrument was used as electronic unit in order to realize a potentially mobile and space-saving setup.

## 2. Results and Discussion

**Figure 1:**
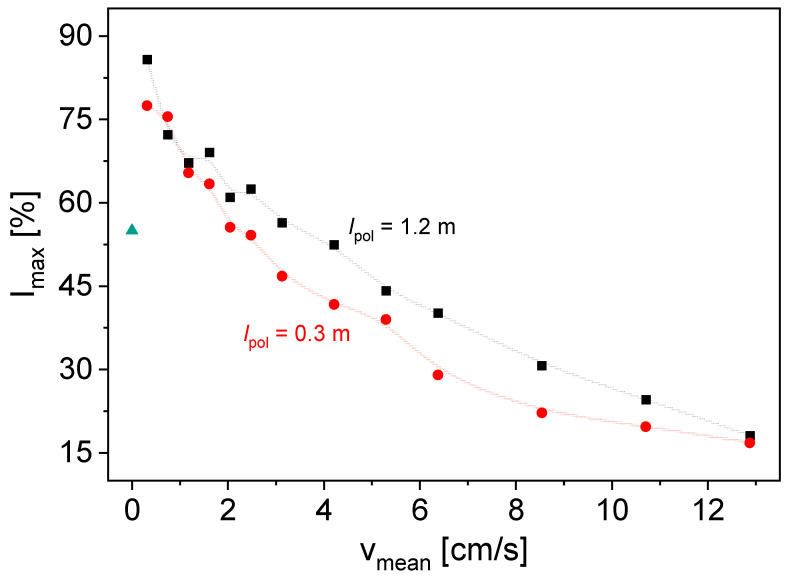
*I*_max_ as a function of *v*_mean_ for a LiCl solution with *c*_LiCl_ = 27.1 g/L and *RD* = 15 s, with *l*_pol_ = 0.3 m (●) and *l*_pol_ = 1.2 m (■). The longer pre-polarization length leads to a larger signal intensity, whereby the dependence of *I*_max_ on *v*_mean_ is roughly the same for both *l*_pol_. B-Splines are guides for the eye. *I*_max_ for *v*_mean_ = 0 cm/s is displayed as ▲.

The maximum signal intensities *I*_max_ of the Hahn echo envelopes are plotted as a function of the mean velocity to show the dependencies on the respective parameters. A value of 100% corresponds to the maximum signal intensity that can be correctly processed by the receiver. For the solution with *c*_LiCl_ = 27.1 g/L and *l*_pol_ = 30 cm, *I*_max_ decreases with increasing *v*_mean_ (Figure 1, red data points). However, for sufficiently small *v*_mean_, *I*_max_ increases compared to the static case of *v*_mean_ = 0 cm/s. This can be explained by the inflow effect, which is well known in flow NMR [20,21]: The flow causes fresh, polarized, but unexcited spins to flow into the sensitive area after previous excitation and echo detection during the repetition time. In the next experiment, their magnetization can be excited, thereby reducing integral saturation effects. This results in a larger integral signal intensity if the repetition time *RD* is smaller than five times the longitudinal relaxation time *T*_1_. For larger *v*_mean_, the residence time in the static magnetic field *B*_0_ along the polarization length of 0.3 m is not sufficient for an almost complete polarization due to the long *T*_1_ of ^7^Li of around 8 s [16]. This results in smaller *I*_max_ values. Please note that *RD* = 15 s in Figure 1 already results in partial saturation in non-flowing liquids and, thus, smaller *I*_max_. For static measurements, *RD* would have to be significantly larger.

The measurements show that the selected experimental setup with the cryogen-free magnet can be used for measurements on ^7^Li, even in flow. The impact of the flow velocity can clearly be derived from the signal intensity as a consequence of the inflow effect, even at the small pre-polarization length. The investigation of the influence of the internal pre-polarization length on the measurement results additionally provides a basis for further potential applications of the magnet. The large bore allows the tube to be wound up in the magnet, increasing *l*_pol_ here to 120 cm.

The signal intensities are larger for the longer pre-polarization length *l*_pol_ = 1.2 m (Figure 1, black data points), which is explained by the larger residence time and the more pronounced longitudinal magnetization build-up. However, the influence of the inflow effect can still be observed. A further increase in the internal *l*_pol_ will result in a larger signal intensity, making the magnet well suited for experiments with the need for pre-polarization, which is especially the case for moieties with long *T*_1_ and low NMR sensitivity.

**Figure 2:**
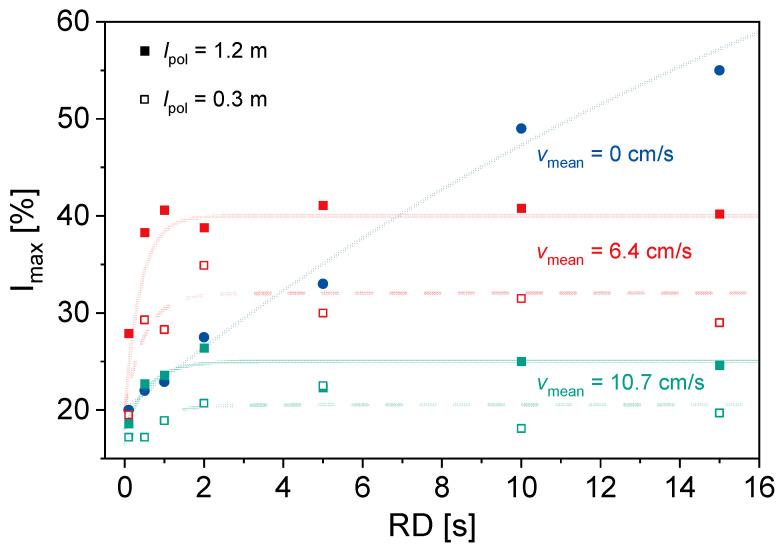
*I*_max_ for *c*_LiCl_ = 27.1 g/L as a function of *RD* for (●) *v*_mean_ = 0 cm/s; (■) *l*_pol_ = 1.2 m, *v*_mean_ = 6.4 cm/s; (□) *l*_pol_ = 0.3 m, *v*_mean_ = 6.4 cm/s; (■) *l*_pol_ = 1.2 m, *v*_mean_ = 10.7 cm/s; and (□) *l*_pol_ = 0.3 m, *v*_mean_ = 10.7 cm/s. Lines are guide for the eyes. *I*_max_ increases with increasing *RD*. A longer pre-polarization length results in a larger *I*_max_. For smaller *RD*, *I*_max_ for *v* = 0 cm/s is between the values of *I*_max_ for *v* > 0 cm/s, and for larger *RD*, *I*_max_ for *v* = 0 cm/s is the largest.

In addition to *v*_mean_, the repetition time *RD* was varied (Figure 2). For *v*_mean_ = 0 cm/s, *I*_max_ increases with increasing *RD*. This is due to saturation effects as a result of the large *T*_1_ of ^7^Li in the used sample. For *v*_mean_ > 0 cm/s, the influence of *l*_pol_ is clearly noticeable. Larger *l*_pol_ leads to larger *I*_max_. Furthermore, a plateau in *I*_max_ occurs as a function of *RD* for *v*_mean_ > 0 cm/s, which is explained by the inflow effect and the smaller influence of saturation effects. At small *RD* and medium *v*_mean_, an increase in *I*_max_ due to the inflow effect can be seen when compared to *v*_mean_ = 0 cm/s. For larger velocities, the shorter residence time in *B*_0_ and smaller pre-polarization lead to reduced *I*_max_. In addition, the outflow effect needs to be considered. The benefit of the described setup becomes obvious in the left part of the graph: *I*_max_ is considerably larger at moderate velocities, even at the largest velocity of 10.7 cm/s, and *I*_max_ is in the range of thermal polarization for the larger pre-polarization length. This observation allows for reducing the repetition time considerably to reasonable values: for example, *RD* around 1 s instead of 8 s for *l*_pol_ = 1.2 m and *v*_mean_ = 6.4 cm/s, which results in eight times faster experiments or a significant gain in the signal-to-noise ratio. The observations are consistent with expectations, making the experimental setup and the selected parameter ranges suitable for these applications.

**Figure 3:**
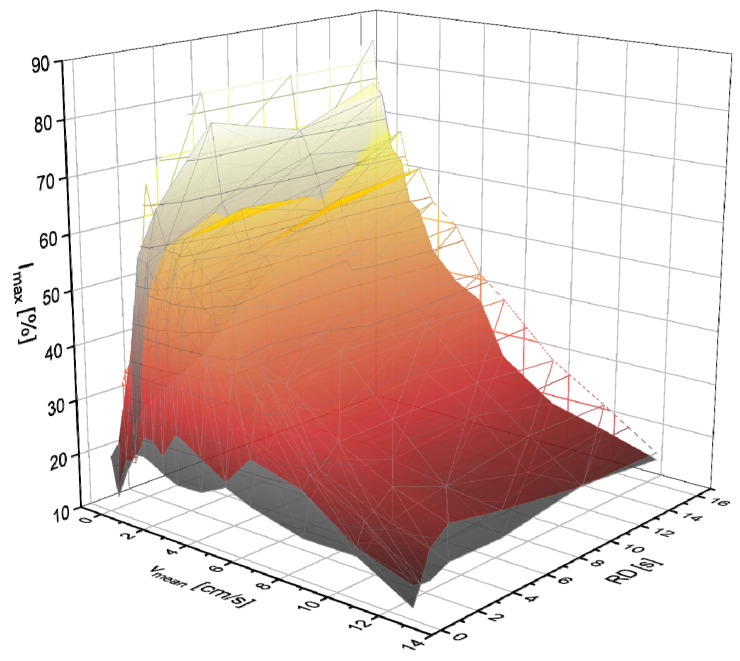
*I*_max_ for *c*_LiCl_ = 27.1 g/L as a function of *v*_mean_ and *RD* for a LiCl solution with *l*_pol_ = 0.3 m (gray scale) and *l*_pol_ = 1.2 m (yellow-red scale).

The three-dimensional (*v*_mean_, *RD*, and *l*_pol_) parameter space was examined using full factorial design so that all inter-dependencies could be identified. The experiments with *c*_LiCl_ = 27.1 g/L show a sufficiently large signal intensity for the investigation of the respective influences for all parameter combinations (Figure 3).

**Figure 4:**
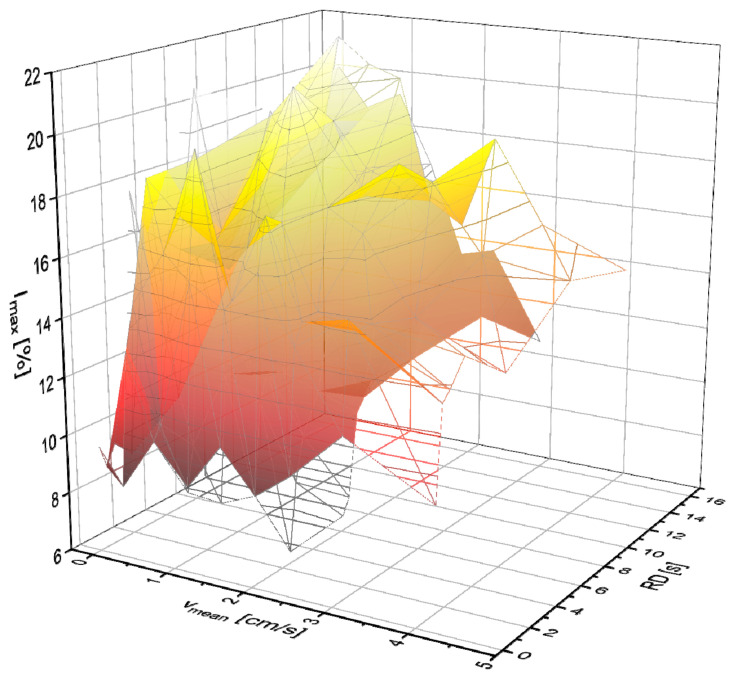
*I*_max_ for *c*_LiCl_ = 15.6 g/L as a function of *v*_mean_ and *RD* for a LiCl solution with *l*_pol_ = 0.3 m (gray scale) and *l*_pol_ = 1.2 m (yellow-red scale).

Spin echoes could also be detected for the aqueous solution of the smaller concentration *c*_LiCl_ = 15.6 g/L. The measurement settings have been adjusted accordingly. *I*_max_ at *v*_mean_ > 4.2 cm/s is barely distinguishable from the noise level, so these data points are not shown (Figure 4).

Also, at smaller concentrations, the positive influence of the longer *l*_pol_ on *I*_max_ is clear. The experiments with smaller *c*_LiCl_ reveal the limitations of the current measurement setup in terms of Li^+^ concentration and flow velocity range.

## 3. Materials and Methods

**Figure 5:**
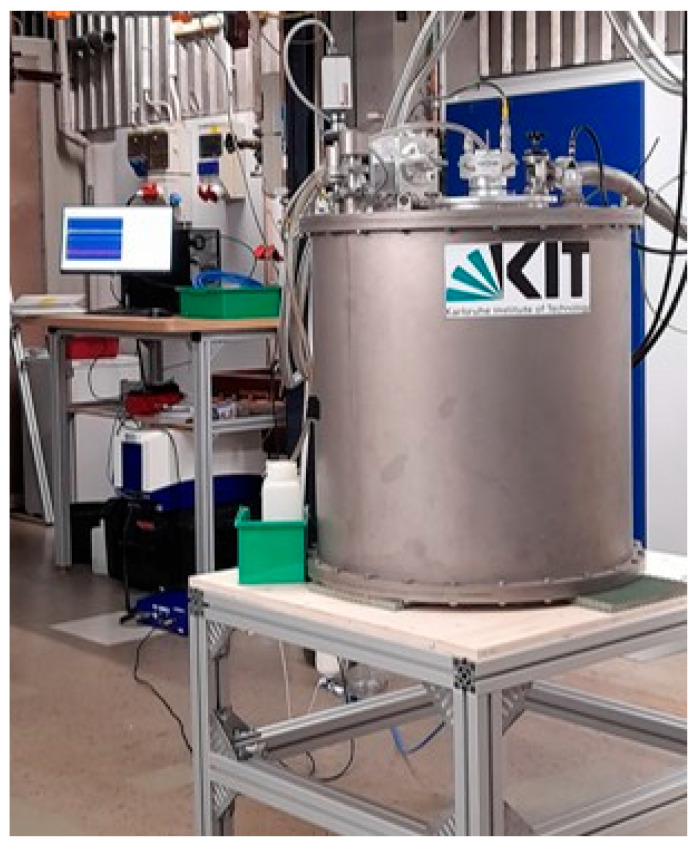
Picture of the magnet on a stand in the front and the NMR control units in the back.

The superconducting magnet used for the investigations consists of a NbTi magnet coil and generates a maximum *B*_0_ of 5 T in a warm vertical bore of 180 mm diameter (Figure 5). The magnet is operated at 4 K and cooled without cryogen using a cryocooler. The cool-down process takes approximately 80 h, whereby only a power supply is required. In the cold state, the maximum field is reached within 42 min. The axial *B*_0_ homogeneity in the relevant area is <1%, and neither cryogenic nor room temperature shim systems are included. Chemical resolution is not the focus of the measurement method as only Li^+^ ions were observed. For compatibility reasons with results on a conventional helium-cooled NMR magnet, a *B*_0_ of 4.7 T was selected, which corresponds to a ^7^Li Larmor frequency of 77.81 MHz. The electronic unit of a Bruker minispec NF series (Bruker BioSpin GmbH & Co. KG, Ettlingen, Germany) was used for control, pulse generation, and data acquisition, whereby the preamplifier was modified in order to extend the frequency range. A Bruker MICWB40 ^7^Li 25 mm LTR micro imaging probe (Bruker BioSpin MRI GmbH, Ettlingen, Germany) was used for the experiments.

The Hahn echo pulse sequence was selected for the measurements [22]. In this pulse sequence, a 90° excitation pulse is followed by a 180° refocusing pulse after the half echo time τ_e_ and then by the detection of a spin echo after a further delay of τ_e_/2. In contrast to single-pulse acquisition, the Hahn echo pulse sequence refocuses the dephasing due to static magnetic field inhomogeneities, making it more suitable for measurements with the magnet used. Since the transverse NMR relaxation properties of the sample were not of interest for the fundamental investigations of this work, measurements were only performed at one echo time so that no echo train was acquired. This enables fast, quantitative measurements of lithium concentration, which is necessary for potential inline applications in quality control and process management.

**Figure 6:**
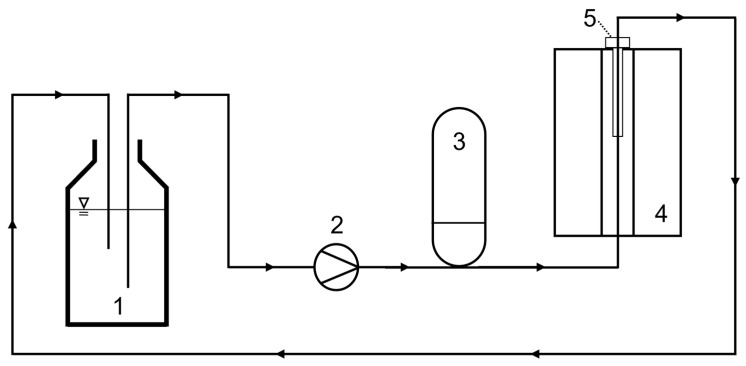
Scheme of the experimental flow setup with the reservoir (1), the peristaltic pump (2), the pressure compensation tank (3), the magnet (4), and the probe (5). The sample is recirculated back to the reservoir.

For the flow-through measurements and to show the principle on the lab scale, a fluidic circuit was built through the magnet. It comprised a reservoir tank, a peristaltic pump (Watson-Marlow 323 S, Watson-Marlow Ltd., Falmouth, UK), and a pressure compensation tank to reduce flow pulsation before the fluid enters the magnet (Figure 6). A tube of polyurethane with an inner diameter of 7 mm was passed through the probe. The flow direction was selected from bottom to top, and the sample was recirculated for longer total experiment times. The probe was positioned in the magnet from the top and held in a reproducible position with a cover plate and precision spacers. A straight, i.e., shortest, possible arrangement of the tube results in a pre-polarization length *l*_pol_ of 30 cm from the lower edge of the magnet to the sensitive area of the probe. To realize a longer pre-polarization length, the tube was wound in a spiral and positioned in the magnet, resulting in *l*_pol_ = 120 cm.

To prove the sensitivity of the measurements on ^7^Li, two aqueous LiCl (Sigma-Aldrich, St. Louis, MO, USA) solutions with concentrations of *c*_LiCl_ = 27.1 g/L and 15.6 g/L were prepared. The solutions were mixed by adding crystalline LiCl to deionized water and shaking the vessel for several minutes. Measurements were made on both solutions in flow at different mean flow velocities *v*_mean_ and repetition times (relaxation delay *RD*). The NMR measurement parameters were determined individually for both concentrations (Table 1).

## 4. Conclusions

The suitability of a cryogen-free magnet at 4.7 T for ^7^Li NMR on flowing samples, for example, in a lithium extraction plant, were investigated. The Hahn echo pulse sequence was used to measure signal intensities and thus make quantitative determinations about lithium concentration. By varying the mean flow velocity and the repetition time, information was obtained about the usability in a process plant and about the influences of the inflow effect on the NMR measurements in the desired area of application. The experiments with two different LiCl concentrations revealed corresponding limitations with regard to the LiCl concentration. The method can therefore be applied in the product streams of a production plant with sufficiently large lithium concentrations. In addition to the suitability of the cryogen-free magnet for ^7^Li NMR measurements, it was also shown that the magnet can be used for internal pre-polarization and that positive effects on the measurement results can be generated. Based on the investigations shown here, measurements could be made on lithium-containing solutions from a real production plant that offers, in addition, considerable paramagnetic relaxation enhancement in the natural composition for the reduction of Li measurement time. This effect could also be used to investigate the influence of other components, particularly those of these paramagnetic constituents. NMR relaxation measurements can also be considered for this purpose in order to gain in-depth insight, also in form of multiple echo sequences. Furthermore, measurements on other NMR-active nuclei and a comprehensive characterization of the pre-polarization properties are conceivable at the larger magnetic fields in comparison to permanent magnets. A further improvement in the measurement process can be achieved by further increasing the polarization length in the magnet. In addition, the sample volume can be increased to further improve the signal-to-noise ratio, which, together with multi-echo sequences, will result in the real inline NMR determination of Li ion content along the enrichment process.

## Figures and Tables

**Table 1:** Measurement and experiment parameters of the flow measurements with the two aqueous LiCl solutions.

Parameter	*c*_LiCl_ = 27.1 g/L	*c*_LiCl_ = 15.6 g/L
Echo time τ_e_ (ms)	1.0	1.0
Repetition time *RD* (s)	0.1 … 15	0.1 … 15
Number of averages (−)	8	16
Receiver gain (dB)	84	88
*v*_mean_ (cm/s)	0 … 12.9	0 … 4.2
*l*_pol_ (cm)	30; 120	30; 120

## Data Availability

The data are available on request from the authors.

## Abbreviations

The following abbreviations are used in this manuscript:

LiCl	Lithium chloride
NMR	Nuclear magnetic resonance
